# Survival and prognosis factors in systemic sclerosis: data of a French multicenter cohort, systematic review, and meta-analysis of the literature

**DOI:** 10.1186/s13075-019-1867-1

**Published:** 2019-04-03

**Authors:** M. R. Pokeerbux, J. Giovannelli, L. Dauchet, L. Mouthon, C. Agard, J. C. Lega, Y. Allanore, P. Jego, B. Bienvenu, S. Berthier, A. Mekinian, E. Hachulla, D. Launay

**Affiliations:** 1 University of Lille, U995 - LIRIC - Lille Inflammation Research International Center, F-59000 Lille, France; 2 INSERM, U995, F-59000 Lille, France; 3 CHU Lille, Département de Médecine Interne et Immunologie Clinique, F-59000 Lille, France; 4 Centre de Référence des Maladies Autoimmunes et Systémiques Rares du Nord et Nord-Ouest de France (CeRAINO), Lille, France; 5 Inserm UMR1167, RID-AGE, Risk Factors and Molecular Determinants of Aging-Related Diseases, Université de Lille, Centre Hosp. Univ Lille, Institut Pasteur de Lille, Lille, France; 6 Service de Médecine Interne, Hôpital Cochin, Centre de Référence pour les Maladies Systémiques Autoimmunes Rares d’Ile de France, Université Paris Descartes, Sorbonne Paris Cité, Assistance Publique-Hôpitaux de Paris (AP-HP), Paris, France; 7 CHU Nantes, Service de Médecine Interne, Nantes, France; 8 Department of Internal and Vascular Medicine, Centre Hospitalier Lyon Sud, Hospices Civils de Lyon, Pierre-Bénite, France; 9 Univ Lyon, UMR 5558, Laboratoire de Biométrie et Biologie Evolutive, CNRS, Claude Bernard University, F-69003 Lyon, France; 10 Hôpital Cochin-APHP-Service de Rhumatologie A, Université Paris Descartes, INSERM U1016, Paris, France; 11 INSERM U 1085 (IRSET), University of Rennes 1, Rennes, France; 12 Service de Médecine Interne CHU Caen, Caen, France; 13 Service de Médecine Interne et Immunologie Clinique, CHU Dijon, Dijon, France; 14 Hôpital Saint-Antoine-APHP-Service de Médecine Interne, Paris, France

**Keywords:** Systemic sclerosis, Prognosis factors, Survival, Meta-analysis

## Abstract

**Background:**

Data on survival and prognosis factors in incident cohorts are scarce in systemic sclerosis (SStc). To describe survival, standardized mortality ratio (SMR), and prognosis factors in systemic sclerosis (SSc), we analyzed a multicenter French cohort of incident patients and performed a systematic review of the literature and meta-analysis.

**Methods:**

A multicenter, French cohort study was conducted between January 1, 2000, and December 31, 2013. Patients were followed-up until July 1, 2016.

A systematic review of the literature was carried out in MEDLINE and EMBASE up to July 2017. Meta-analysis was performed using all available data on SMR and hazard ratios of prognosis factors.

**Results:**

A total of 625 patients (493 females, 446 lcSSc) were included. During the study period, 104 deaths (16.6%) were recorded and 133 patients were lost to follow-up. Overall survival rates at 1, 3, 5, and 10 years from diagnosis were 98.0%, 92.5%, 85.9%, and 71.7% respectively in the French cohort. Overall SMR was 5.73 (95% CI 4.68–6.94). Age at diagnosis > 60 years, diffuse cutaneous SSc, scleroderma renal crisis, dyspnea, 6-min walking distance (6MWD), forced vital capacity < 70%, diffusing capacity of the lungs for carbon monoxide < 70%, pulmonary hypertension (PH), telangiectasia, valvular disease, malignancy, anemia, and CRP > 8 mg/l were associated with a poorer survival after adjustment.

Eighteen studies (11,719 patients) were included in the SMR meta-analysis and 36 studies (26,187 patients) in the prognosis factor analysis. Pooled SMR was 3.45 (95%CI 3.03–3.94). Age at disease onset, male sex, African origin, diffuse cutaneous SSc, anti-Scl70 antibodies, cardiac and renal involvement, interstitial lung disease, PH, and malignancy were significantly associated with a worse prognosis. Anti-centromere antibodies were associated with a better survival.

**Conclusions:**

Overall, our study highlights a high mortality rate in SSc patients and confirms previously described prognosis factors related to skin extension and organ involvement while identifying additional prognosis factors such as autoantibody status, telangiectasia, 6MWD, and valvular disease.

**Electronic supplementary material:**

The online version of this article (10.1186/s13075-019-1867-1) contains supplementary material, which is available to authorized users.

## Background

Systemic sclerosis (SSc) is an autoimmune disease, characterized by microvascular damage, dysregulation of both innate and adaptative immunity, and fibrosis of multiple organs. The causes of SSc-related deaths evolved over the last decades, with cardiac and respiratory complications currently being the leading causes of death [1, 2].

Prior cohort studies comparing contemporary and historical cohort have suggested an improvement of survival rates over time [1, 3, 4]. Yet, two recent meta-analyses have reported that standardized mortality ratio (SMR) was stable over time [5, 6]. Moreover, most of the observational studies investigating mortality in SSc included prevalent cases, which may result in an underestimation of mortality due to a survivor bias. Data on survival in incident cohorts are scarce in SSc [7–12].

Previous studies [7, 13–26] reported risk factors for poor survival in SSc such as male sex, diffuse cutaneous subtype, and specific organ involvement. Recently, Elhai et al. developed a prognostic score from the large EUSTAR database, which accurately predicts 3-year mortality [2]. To our knowledge, two meta-analyses combined the results of the available literature to assess prognosis factors [5, 27]. However, these meta-analyses did not assess prognosis factors such as auto-antibody profile, genetic background, and cancer and did not assess the influence of prevalent versus incident cases.

The aim of the present study was to fill these gaps by assessing survival and prognosis factors in a multicenter French cohort of incident SSc patients and by performing a systematic review of the literature and meta-analysis including all available prognosis factors and SMR.

## Methods

### Population

The French National Scleroderma Cohort includes 42 centers. The present analysis was restricted to five university hospitals, Lille, Paris (two centers), Nantes, and Lyon to ensure better quality of data, especially on survival data. These five centers participated in recruiting about two thirds of the National Cohort. Data were retrospectively collected before 2010 and then prospectively collected.

Patients were included between January 1, 2000, and December 31, 2013, if they met the following inclusion criteria: (i) be aged over 18, (ii) fulfill the ACR 1980 preliminary classification criteria [28] or ACR/EULAR 2013 classification criteria [29] for SSc, (iii) have at least one additional visit after the inclusion visit, and (iv) be incident cases, defined as patients having disease duration from time of diagnosis to enrolment in the study of less than 3 years. Patients were followed-up until July 1, 2016. Patients were considered as lost to follow-up if the vital status could not be ascertained. When possible, the vital status was ascertained by querying death registers at birth town councils.

### Collected data and variable definition

Data collected at the inclusion visit were patient demographics, history of Raynaud phenomenon (RP) and first non-RP symptom, SSc subtype and modified Rodnan skin score (mRSS), auto-antibody profile, and organ involvement.

Disease onset was defined as the time of onset of first non-RP symptom. Interstitial lung disease was diagnosed on HRCT or chest x-ray. Pulmonary function tests including forced vital capacity (FVC) and diffusing capacity of the lungs for carbon monoxide (DLCO) were collected. Six-minute walking distance (6MWD) was collected. Pulmonary hypertension (PH) was suspected on a Doppler echocardiogram when systolic pulmonary arterial pressure (PAP) was estimated to be > 35 mmHg or maximum tricuspid regurgitant jet velocity > 2.8 m/s. Pulmonary arterial hypertension (PAH) was confirmed by right heart catheterization (RHC) when mean PAP was found to be ≥ 25 mmHg at rest, with mean pulmonary arterial wedge pressure ≤ 15 mmHg. EKG alterations, left ventricular ejection fraction (LVEF), diastolic dysfunction [30], valvular disease (excluding tricuspid valve regurgitation to avoid confounding with PH), and pericarditis were recorded according to the American Society of Echocardiography and European Association of Cardiovascular Imaging guidelines [30]. Scleroderma renal crisis was defined as new onset hypertension > 150/85 mmHg associated with a decrease in renal function or manifestations of malignant hypertension. Gastrointestinal tract involvement included reflux, dysmotility, constipation, or diarrhea; signs of bacterial overgrowth and/or malabsorption; and abnormal manometry and/or endoscopy test. Muscle involvement included myalgia and/or muscle weakness and/or elevation of creatinine kinase (CPK). Joint involvement included arthralgia, synovitis, and/or tendon friction rubs. Anemia was defined as a hemoglobin level < 12 g/dl. Smoking included self-reported current or former cigarette smoking.

### Systematic review and meta-analysis

The meta-analysis was conducted according to MOOSE guidelines [31]. MEDLINE and EMBASE databases were queried by two of the authors (MRP and DL) using the following search terms: ((systemic sclerosis [Title]) OR (scleroderma, systemic[Title])) AND ((death) OR (mortality) OR (prognosis) OR (survival)). Cochrane did not retrieve additional abstracts. All records published before July 1, 2017, were included in the search. Language was restricted to English or French. Reference list of selected studies was hand-searched for additional relevant studies to be included in the meta-analysis.

Two of the authors (MRP and DL) independently screened the titles and abstracts of the retrieved records to identify eligible articles. The full text of eligible articles was read for inclusion in the meta-analysis. Selected articles were compared, and in case of disagreement, decisions were made by consensus.

Cohort studies of unselected adult SSc patients assessing routine clinical and laboratory prognosis factors and SMR were included. Studies which included patients diagnosed with SSc overlap with other connective tissue diseases were excluded.

Studies from the same centers were included if their respective study periods were different. If, for the same center, two studies covered an overlapping study period, data from the largest cohort were kept in the analysis. A study was recorded as an incident according to the authors’ definition.

Quality of the studies was assessed using the Newcastle-Ottawa scale [32].

Data were extracted and entered into a predefined spreadsheet table which included the following items: study design, length of follow-up, definition of disease onset, disease duration, SMR, and adjusted or, if unavailable, unadjusted hazard ratios (HR) for each studied prognosis factor.

### Data analysis

Characteristics of the population were described using mean ± standard deviation or median (interquartile range (IQR)) in case of non-normality, for quantitative variables, and number (percentage) for qualitative variables. Comparisons between limited cutaneous SSc (lcSSc) and diffuse cutaneous SSc (dcSSc) patients were conducted using the Student *t* test or Wilcoxon test in case of non-normality for quantitative variables and Fisher’s exact test for qualitative variables.

Survival was estimated from diagnosis using the Kaplan-Meier method. Prognosis factors were assessed by Cox regression analysis in the non-adjusted analysis and subsequently adjusted for age, sex, and SSc subtype. The assumption that hazard ratios were constant over time was verified. SMR was calculated as the ratio of observed death in the cohort to the number of death of the French age/sex-matched population in 2014.

We calculated weighted pooled summary estimates of SMR and HR of prognosis factors. For each meta-analysis, we used the DerSimonian and Laird method. Accordingly, studies were considered to be a random sample from a population of studies. Heterogeneity was assessed using an *I*^2^ statistic and a chi-square heterogeneity statistic. A random-effects model was used to combine data. The overall effect was estimated using a weighted average of individual effects, with weights inversely proportional to variance in observed effects. Publication bias was evaluated with a funnel plot and Egger’s test. The pooled SMR and HR were estimated with 95% confidence interval (CI). Meta-regression was used to assess the impact of mid-cohort year, the proportion of males, the proportion of diffuse cutaneous forms, and the prevalence of anti-Scl70 antibodies on SMR. The impact of diagnosis of PH by RHC on the association of PH with mortality was evaluated. Separate analyses were performed for (i) SMR according to whether a given study included incident cases only and (ii) HR of PH diagnosed by either echocardiography and/or RHC and PH diagnosed by RHC.

All analyses were performed using R software with the survival and metafor packages. *p* values less than 0.05 were considered significant.

## Results

### French cohort study

#### Baseline characteristics

A total of 625 patients (493 females, 446 lcSSc) were included. Mean age at disease onset was 52.7 ± 14.9 years. The median disease duration from disease onset was 0.8 (IQR 2.2) years. Median follow-up time was 4.4 (IQR 5.3) years. The baseline characteristics are shown in Table 1.

**Table 1:** Demographics and clinical characteristics of 625 patients with SSc at baseline

	*N* (*N* for dcSSc)	Total	dcSSc	lcSSc	*p*
Demographics					
Female sex	625/179	493 (79)	124 (69)	369 (83)	< 0.001
Age at first RP (years)	554/155	45.4 ± 15.7	45.8 ± 15.7	45.3 ± 15.8	0.736
Age at first non-RP symptom (years)	502/160	50.6 ± 14.5	48.5 ± 14.4	51.5 ± 14.4	0.031
Age at diagnosis (years)	625/179	52.7 ± 14.9	49.5 ± 14.5	53.9 ± 14.9	< 0.001
Disease duration from first non-RP symptom to diagnosis (years)	499/160	0.8 [2.2]	0.7 [1.4]	0.9 [2.7]	0.042
Follow-up time from inclusion to death or last visit (years)	625/179	4.4 [5.3]	4.0 [5.2]	4.8 [5.3]	0.023
Genetic background					
European	503/147	453 (90)	118 (80)	335 (94)	< 0.001
African	503/147	50 (10)	29 (20)	21 (6)	< 0.001
Skin involvement					
lcSSc	625/179	446 (71)	–	–	
mRSS	342/123	9.2 ± 10.2	19.6 ± 10.1	3.5 ± 3.6	< 0.001
Telangiectasia	572/160	264 (46)	65 (41)	199 (48)	0.112
Calcinosis	549/152	64 (12)	7 (5)	57 (14)	< 0.001
Digital ulcers (past or active)	538/145	161 (30)	66 (46)	95 (24)	< 0.001
Pulmonary involvement					
NYHA					0.702
Classes I–II	515/150	425 (83)	122 (81)	303 (83)	
Classes III–IV	515/150	90 (17)	28 (19)	62 (17)	
6MWD (meters)	274/61	427 ± 127	432 ± 135	425 ± 125	0.705
TLC < 70% predicted	472/145	64 (14)	33 (23)	31 (9)	< 0.001
FVC < 70% predicted	475/148	82 (17)	44 (30)	38 (12)	< 0.001
DLCO < 70% predicted	471/141	249 (53)	102 (72)	147 (45)	< 0.001
Interstitial lung disease	582/166	262 (45)	115 (69)	147 (35)	< 0.001
PH (echo. and/or RHC)	547/157	67 (12)	18 (11)	49 (13)	0.775
sPAP (echo.)					0.004
< 35 mmHg	397/118	307 (77)	89 (75)	218 (78)	
35–46 mmHg	397/118	43 (11)	21 (18)	22 (8)	
> 46 mmHg	397/118	47 (12)	8 (7)	39 (14)	
PAH (RHC)	490/116	40 (8)	4 (3)	36 (10)	0.033
Heart involvement					
Arrhythmia	519/150	17 (3)	5 (3)	12 (3)	1.000
AV block	512/146	7 (1)	4 (3)	3 (1)	0.106
BB block	479/128	16 (3)	6 (5)	10 (3)	0.388
LVEF (%)	402/102	64.9 ± 7.1	65.5 ± 8.6	64.7 ± 6.6	0.251
Diastolic dysfunction	423/110	20 (5)	6 (5)	14 (4)	0.613
Pericarditis	478/136	32 (7)	14 (10)	18 (5)	0.066
Valvular disease	430/111	29 (7)	5 (5)	24 (8)	0.380
Renal involvement					
GFR < 80 ml/min	459/136	179 (39)	38 (28)	141 (44)	0.002
Scleroderma renal crisis	428/139	44 (10)	31 (22)	13 (5)	< 0.001
Gastrointestinal involvement	611/172	429 (70)	135 (78)	294 (67)	0.006
BMI (kg/m^2^)	514/159	24.4 ± 5.0	23.6 ± 4.0	24.7 ± 5.3	0.016
Albuminemia < 35 g/l	331/108	52 (16)	28 (26)	24 (11)	< 0.001
Muscular involvement	604/172	137 (23)	71 (41)	66 (15)	< 0.001
CPK > 200 IU/l	250/82	66 (26)	33 (40)	33 (20)	< 0.001
Joint involvement	598/172	291 (49)	127 (74)	164 (39)	< 0.001
Cancer	625/179	49 (8)	17 (10)	32 (7)	0.327
Hemoglobin	559/163	13.0 ± 1.6	12.5 ± 1.6	13.1 ± 1.5	< 0.001
Anemia	559/163	127 (23)	52 (32)	75 (19)	0.001
CRP > 8 mg/l	470/136	118 (25)	57 (42)	61 (18)	< 0.001
Serologic features					
ACA	557/151	221 (40)	6 (4)	215 (53)	< 0.001
Anti-Scl70 antibodies	504/149	177 (35)	90 (60)	87 (25)	< 0.001
Anti-U1RNP antibodies	342/63	15 (4)	4 (6)	11 (4)	0.492
Anti-RNAP3 antibodies	345/72	18 (5)	13 (18)	5 (2)	< 0.001
Anti-PMScl antibodies	343/62	16 (5)	3 (5)	13 (5)	1.000
Anti-SSa antibodies	387/79	60 (16)	20 (25)	40 (13)	0.014
Anti-SSb antibodies	338/62	11 (3)	3 (5)	8 (3)	0.431
APL antibodies	441/129	31 (7)	16 (12)	15 (5)	0.007
Low complement	482/130	18 (4)	6 (5)	12 (3)	0.589
Smoking	572/158	215 (38)	69 (44)	146 (35)	0.067

Results are expressed as *n* (%) for qualitative variables and mean ± SD or median [IQR] for quantitative variables

*N* number of patients with available data, *lcSSc* limited cutaneous systemic sclerosis, *mRSS* modified Rodnan score, *GFR* glomerular filtration rate, *AV block* atrioventricular block, *BB block* bundle branch block, *LVEF* left ventricular ejection fraction, *PH* pulmonary hypertension, *PAH* pulmonary arterial hypertension, *echo* echocardiography, *RHC* right heart catheterization, *6MWD* 6-min walking distance, *sPAP* systolic pulmonary arterial pressure, *TLC* total lung capacity, *FVC* forced vital capacity, *DLCO* diffusing capacity of the lungs for carbon monoxide, *CRP* C reactive protein, *BMI* body mass index, *ACA* anti-centromere antibodies, *APL* antiphospholipid antibodies

### Survival and standardized mortality ratio

**Fig. 1:**
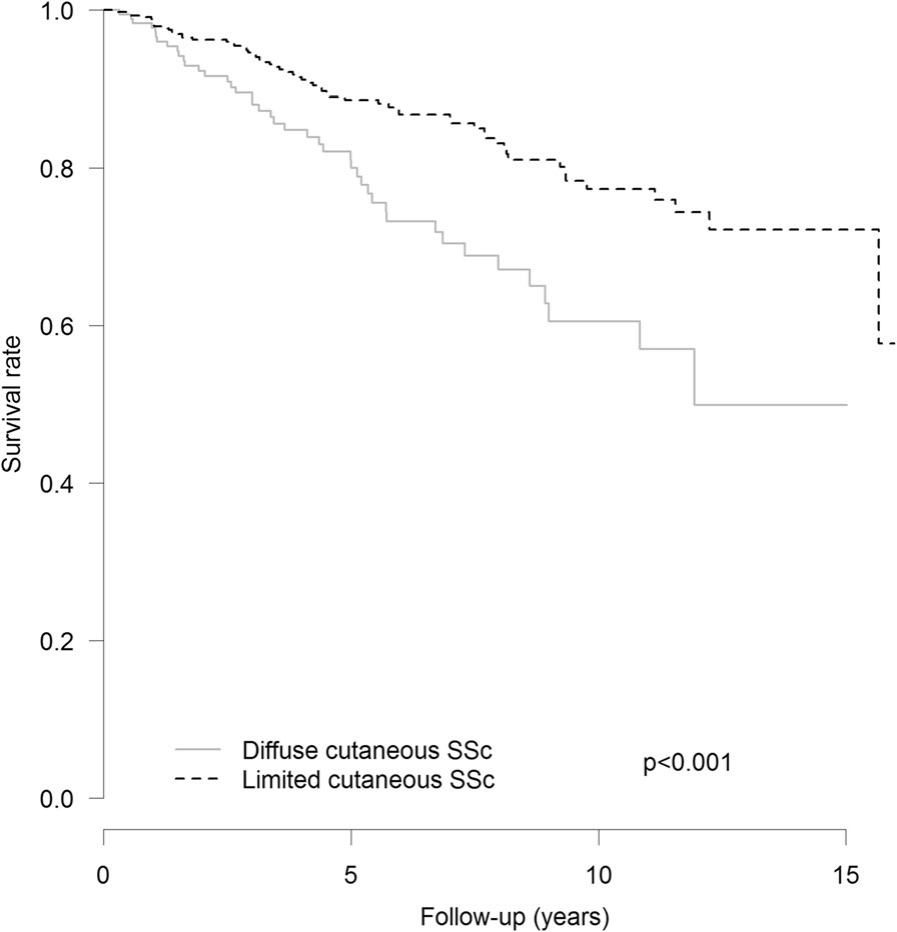
Kaplan-Meier survival curves from diagnosis for lcSSc and dcSSc patients in the French cohort

During the study period, 104 deaths (16.6%) were recorded and 133 patients were lost to follow-up. Overall survival rates at 1, 3, 5, 10, and 15 years from diagnosis were 98.0% (95% CI 96.9–99.1%), 92.5% (90.4–94.7%), 85.9% (82.8–89.1%), 71.7% (66.3–77.5%), and 53% (33.8–83.4%) respectively. Survival rates for the diffuse and limited cutaneous subtypes are shown in Fig. 1 and in Additional file 1: Table S1. Overall SMR was 5.73 (95% CI 4.68–6.94).

### Prognosis factors

Age of diagnosis > 60 years, dcSSc subtype, telangiectasia, scleroderma renal crisis, severe dyspnea NYHA functional classes III and IV, a shorter distance at the 6MWD, FVC < 70%, DLCO < 70%, PH, valvular disease, anemia, CRP > 8 mg/l, and cancer were associated with a worse prognosis (Table 2).

**Table 2:** Prognosis factors: non-adjusted and adjusted analysis on age at diagnosis, sex, and SSc subtype in the French cohort

	Non-adjusted HR	*p*	Adjusted HR	*p*
Demographics				
Male sex	2.00 (1.31–3.05)	0.001	1.53 (0.98–2.39)	0.060
Age at diagnosis (per 1 year)	1.05 (1.04–1.07)	< 0.001	1.08 (1.04–1.12)	< 0.001
Age at diagnostic > 60 years	4.97 (2.53–9.78)	< 0.001	5.79 (2.92–11.49)	< 0.001
Disease duration at time of diagnosis (per 1 year)	1.02 (0.97–1.07)	0.542	1.01 (0.96–1.06)	0.763
African origin (vs. European)	0.79 (0.38–1.62)	0.516	0.93 (0.43–2.03)	0.864
Skin involvement				
dcSSc subtype (vs. lcSSc)	2.06 (1.39–3.05)	< 0.001	2.40 (1.58–3.64)	< 0.001
mRSS > 5	1.24 (1.12–1.38)	< 0.001	1.21 (1.03–1.43)	0.022
Past and/or active digital ulcers	1.22 (0.79–1.90)	0.371	1.29 (0.81–2.04)	0.277
Telangiectasia	1.64 (1.08–2.48)	0.019	1.55 (1.02–2.35)	0.039
Calcinosis	1.37 (0.79–2.36)	0.260	1.22 (0.69–2.16)	0.503
Lung involvement				
NYHA class I	–		–	
NYHA class II	2.68 (1.46–4.92)	0.001	2.37 (1.29–4.36)	0.006
NYHA class III	17.53 (3.97–14.27)	< 0.001	6.74 (3.53–12.88)	< 0.001
NYHA class IV	25.76 (10.55–62.92)	< 0.001	16.61 (6.68–41.26)	< 0.001
NYHA classes III–IV (vs. class I)	4.68 (3.07–7.13)	< 0.001	4.33 (2.82–6.66)	< 0.001
6MWD (per 100 m)	0.46 (0.36–0.58)	< 0.001	0.51 (0.39–0.67)	< 0.001
TLC < 70% predicted	3.87 (2.36–6.35)	< 0.001	3.38 (1.96–5.82)	< 0.001
FVC < 70% predicted	3.11 (1.92–5.02)	< 0.001	2.79 (1.62–4.80)	< 0.001
DLCO < 70% predicted	4.01 (2.33–6.89)	< 0.001	3.31 (1.87–5.88)	< 0.001
Interstitial lung disease	1.99 (1.32–2.99)	< 0.001	1.50 (0.96–2.34)	0.072
PH (echo. and/or RHC)	5.01 (3.18–7.89)	< 0.001	4.15 (2.59–6.65)	< 0.001
sPAP < 35 mmHg	–		–	
35–46 mmHg	2.05 (0.98–4.28)	0.056	1.26 (0.58–2.70)	0.559
> 46 mmHg	6.44 (3.69–11.22)	< 0.001	5.94 (3.30–10.72)	< 0.001
PAH (RHC)	4.96 (2.82–8.72)	< 0.001	4.39 (2.43–7.93)	< 0.001
Heart involvement				
Arrhythmia	2.44 (0.98–6.02)	0.054	1.31 (0.52–3.32)	0.569
AV block	0.95 (0.13–6.80)	0.956	1.15 (0.15–8.58)	0.890
BB block	1.26 (0.31–5.15)	0.748	1.37 (0.33–5.67)	0.661
LVEF < 50%	1.82 (0.25–13.24)	0.555	0.92 (0.12–6.84)	0.938
Diastolic dysfunction	1.36 (0.43–4.35)	0.603	0.97 (0.30–3.13)	0.953
Pericarditis	1.74 (0.84–3.61)	0.139	1.07 (0.50–2.26)	0.864
Valvular disease	4.03 (1.97–8.25)	< 0.001	2.20 (1.05–4.60)	0.037
Renal involvement				
Scleroderma renal crisis	3.44 (2.01–5.89)	< 0.001	2.95 (1.61–5.40)	< 0.001
GFR < 80 ml/min	1.64 (1.06–2.52)	0.025	1.37 (0.85–2.21)	0.199
Gastrointestinal involvement	1.07 (0.68–1.69)	0.756	1.02 (0.65–1.62)	0.916
BMI < 18.5 kg/m2	1.10 (0.45–2.74)	0.831	1.79 (0.71–4.51)	0.220
Albuminemia < 35 g/l	2.30 (1.24–4.30)	0.009	1.45 (0.75–2.82)	0.270
Muscular involvement	1.66 (1.10–2.51)	0.016	1.46 (0.92–2.31)	0.106
CPK > 200 IU/L	1.27 (0.58–2.76)	0.550	1.15 (0.50–2.64)	0.740
Joint involvement	1.22 (0.82–1.80)	0.329	1.08 (0.70–1.66)	0.720
Cancer	2.44 (1.41–4.21)	0.001	1.86 (1.07–3.26)	0.029
Anemia	2.66 (1.75–4.06)	< 0.001	2.37 (1.54–3.66)	< 0.001
CRP > 8 mg/l	2.05 (1.28–3.27)	0.003	1.70 (1.02–2.82)	0.041
Serologic features				
ACA	0.95 (0.62–1.44)	0.795	0.85 (0.55–1.31)	0.459
Anti-Scl70 antibodies	0.87 (0.55–1.36)	0.534	0.82 (0.51–1.30)	0.390
Anti-U1RNP antibodies	1.41 (0.51–3.93)	0.506	1.32 (0.44–3.92)	0.616
Anti-RNAP3 antibodies	0.96 (0.23–3.94)	0.949	1.32 (0.44–3.92)	0.616
Anti-PMScl antibodies	0.33 (0.05–2.41)	0.277	0.49 (0.07–3.54)	0.476
APL antibodies	1.54 (0.71–3.35)	0.280	1.18 (0.53–2.63)	0.679
Low complement	2.40 (0.97–5.95)	0.059	2.38 (0.95–5.95)	0.063
Smoking	1.06 (0.69–1.62)	0.795	0.97 (0.59–1.59)	0.901

Results are expressed as hazard ratios and 95% confidence interval

*lcSSc* limited cutaneous systemic sclerosis, *dcSSc* diffuse cutaneous systemic sclerosis, *mRSS* modified Rodnan score, *GFR* glomerular filtration rate, *AV block* atrioventricular block, *BB block* bundle branch block, *LVEF* left ventricular ejection fraction, *PH* pulmonary hypertension, *PAH* pulmonary arterial hypertension, *echo* echocardiography, *RHC* right heart catheterization, *6MWD* 6-min walking distance, *sPAP* systolic pulmonary arterial pressure, *HRCT* high-resolution computer tomography, *TLC* total lung capacity, *FVC* forced vital capacity, *DLCO* diffusing capacity of the lungs for carbon monoxide, *CRP* C reactive protein, *BMI* body mass index, *ACA* anti-centromere antibodies, *APL* antiphospholipid antibodies

No association was found for digital ulcers, gastrointestinal, articular, muscular involvement, and specific auto-antibodies after adjustment.

Male sex showed a trend towards worse outcome, but without reaching statistical significance (HR = 1.53; 95% CI 0.98–2.39; *p* = 0.06).

### Meta-analysis: study selection

**Fig. 2:**
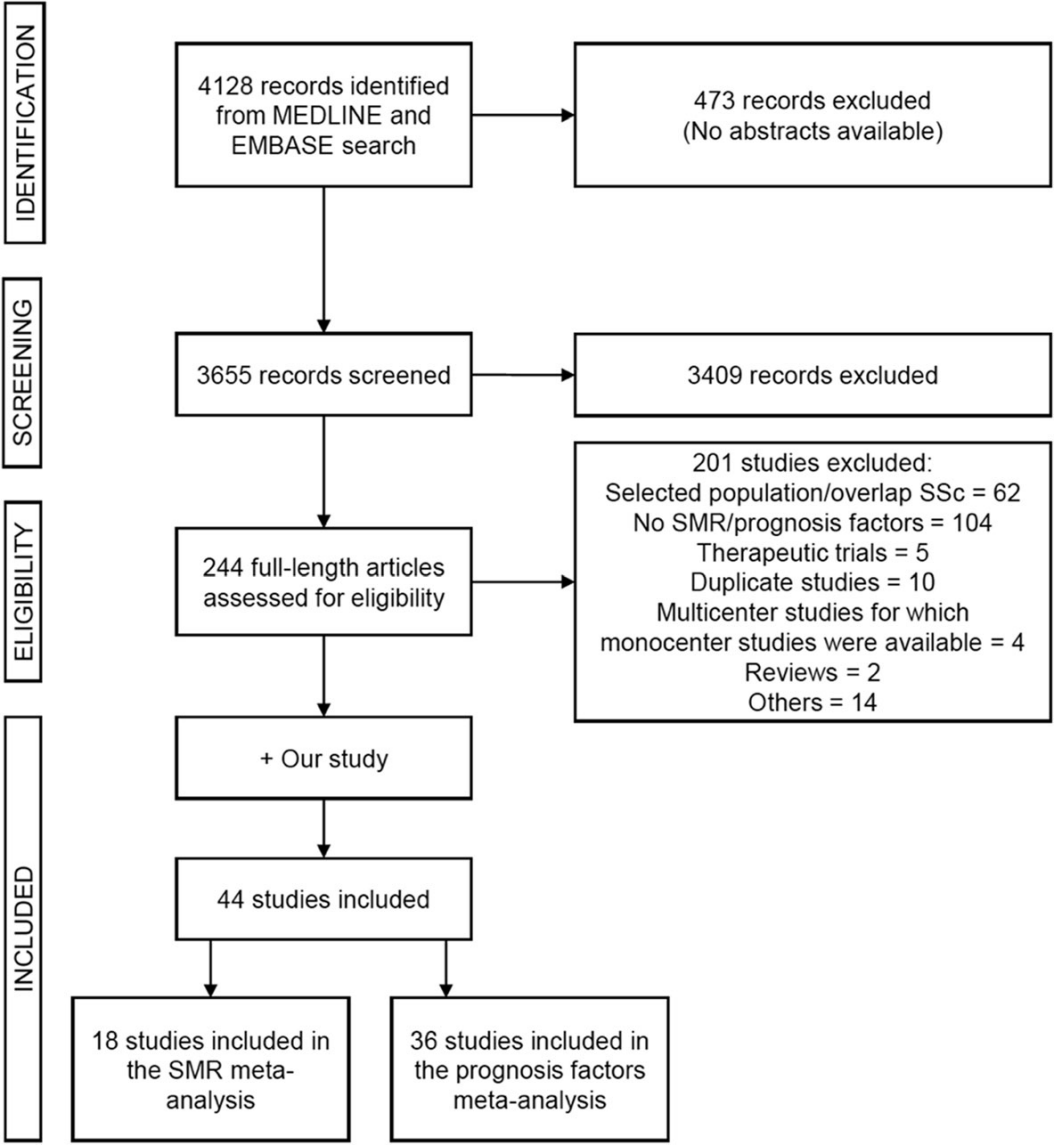
Flow chart showing search strategy to identify studies in the meta-analysis

A total of 4128 citations were assessed for inclusion. After screening, 244 abstracts were deemed potentially relevant and the full-text copies were obtained. Of these articles, 44 studies, including our cohort, were included in the meta-analysis (Fig. 2). Eighteen articles were included in the SMR analysis, representing a total population of 11,719 patients [7–12, 15, 22–24, 26, 33–38]. Thirty-six studies were included in the prognosis factor analysis, representing a total of 26,187 patients [3, 7, 10–18, 20, 22–26, 34, 39–55]. No study was excluded based on poor quality. The main characteristics of the studies are summarized in Additional file 1: Table S2 and Table S3.

### SMR meta-analysis

The pooled SMR for all studies was 3.45 (95% CI 3.03–3.94; *I*^*2*^ = 88.8%; *p*(het) < 0.001). The pooled SMR for studies including only incident patients was 3.64 (95% CI 3.06–4.34; *I*^2^ = 82.0%; *p*(het) < 0.001), and the pooled SMR for studies including prevalent patients was 3.28 (95% CI 2.69–3.99; *I*^2^ = 91.6%; *p*(het) < 0.001). There was no funnel plot asymmetry, and Egger’s test failed to provide any evidence for small study effect, making publication bias unlikely. Meta-regression stratified by study type (incident or prevalent) did not show any association with SMR (*p* = 0.461), meaning no statistical difference between pooled SMR of incident and prevalent studies. Subsequent analyses were therefore conducted on all studies. Meta-regression analysis revealed a significant increase of SMR with proportion of dcSSc (*p* < 0.001) and prevalence of anti-Scl70 antibodies (*p* = 0.021). There was no association with male sex (*p* = 0.130). There was no significant association between SMR and mid-cohort year (*p* = 0.656) (Additional file 2: Figure S1. Prognosis factors meta-analysis).

### Prognosis factor meta-analysis

Table 3 shows the results of the meta-analysis of prognosis factors. Age at disease onset, age at diagnosis, male sex, African origin, dcSSc, anti-Scl70 antibodies, renal involvement, scleroderma renal crisis, ILD, cardiac involvement, PH, and cancer were significantly associated with a worse prognosis. The presence of PH, diagnosed by Doppler echocardiography and/or RHC, was associated with a poor outcome (pooled HR = 3.44; 95% CI 2.59–4.58; *I*^2^ = 61.5%; *p*(het) = 0.002). Meta-analysis of the five studies with PH defined by RHC revealed a pooled HR of 5.27 (95% CI 2.98–9.31; *I*^2^ = 63.7%; *p*(het) = 0.027) for mortality. Heterogeneity could not be fully explained by the use of either echocardiography or RHC alone in defining PH as revealed by meta-regression stratified by the PH diagnosis method (*p* for residual heterogeneity = 0.012). The presence of ACA was associated with a better survival, while the presence of joint involvement was not associated with prognosis (Additional file 2: Figure S2).

**Table 3:** Results of the meta-analysis of prognosis factors in SSc

	Number of cohorts	HR	95% CI	*I*^2^ (%)	*p*(het)	Egger’s test
Age at disease onset (per 1 year)	6	1.05	(1.04–1.07)	68.6	0.007	0.783
Age at diagnosis (per 1 year)	5	1.04	(1.04–1.05)	71.2	0.008	0.025
Male sex	21	1.87	(1.61–2.18)	50.9	0.004	< 0.001
African origin	5	1.38	(1.15–1.66)	25.0	0.255	0.774
dcSSc	23	1.90	(1.62–2.23)	58.3	< 0.001	< 0.001
Anti-Scl70 autoantibodies	13	1.38	(1.09–1.74)	49.6	0.022	0.024
ACA	8	0.62	(0.47–0.82)	56.4	0.025	0.590
Joint involvement	4	1.32	(0.82–2.12)	54.0	0.089	0.508
Renal involvement	9	2.79	(1.95–3.99)	50.9	0.039	0.512
Scleroderma renal crisis	10	3.89	(2.38–6.36)	75.6	< 0.001	0.097
ILD	14	2.34	(1.78–3.08)	69.5	< 0.001	< 0.001
Cardiac involvement	7	4.35	(2.28–8.29)	89.9	< 0.001	0.077
PH (echocardiography or RHC)	13	3.44	(2.59–4.58)	61.5	0.002	0.057
PH (RHC)	5	5.27	(2.98–9.31)	63.7	0.027	0.761
Cancer	6	2.11	(1.27–3.50)	76.2	< 0.001	0.016

Results are expressed as hazard ratios with 95% confidence interval. The *I*^2^ statistics describes the percentage of variation across studies that is due to heterogeneity rather than chance. *p*(het) is the *p* value for the *휒*^2^ test for heterogeneity. Egger’s test checks for funnel plot asymmetry

*dcSSc* diffuse cutaneous systemic sclerosis, *ILD* interstitial lung disease, *ACA* anti-centromere antibodies, *PH* pulmonary hypertension, *RHC* right heart catheterization

## Discussion

The main results of our study are (i) a high risk of mortality in our cohort of incident patients, as shown by a high SMR of 5.73; (ii) the identification of age > 60 years, dcSSc, dyspnea, PH, low FVC, low DLCO, kidney involvement, valvular disease, cancer, telangiectasia, shorter 6MWD, anemia, and inflammation as prognosis factors in our cohort; (iii) a high pooled SMR of 3.45 in the meta-analysis of the literature, including our new cohort; and (iv) the additional identification of male sex, African origin, ILD, cardiac involvement, and anti-Scl-70 antibodies as associated with worse prognosis in our meta-analysis, while ACA were associated with better prognosis.

### Survival and SMR

With a mid-cohort year of 2008, our study population is the largest multicenter incident and well-phenotyped cohort study of SSc patients in France and is among the most recent published to date in the literature. The overall survival rates at 5 and 10 years from diagnosis were 85.9% and 71.7%, respectively, and are lower than those reported in other recent cohorts [7, 22, 24, 44, 54]. We also report one of the highest SMR of 5.73. These differences could be explained by a high heterogeneity between studies as well as methodological issues such as the inclusion of prevalent cases in many studies or differences in time origin from which survival time is calculated (from disease onset, diagnosis, or enrolment). It is usually admitted that studies including prevalent cases underestimate mortality and that better survival is observed in prevalent patients with longer disease duration prior to inclusion. Yet, our meta-analysis did not show a significant difference between pooled SMR of studies that included prevalent cases and those restricted to incident according to the authors’ definition. The high heterogeneity observed within studies with incident patients could be due to the definition of incidence and the proportion of males and patients with anti-Scl70 antibodies. This high heterogeneity could explain the lack of difference between incident and prevalent cohorts. Meta-regression showed a significant association between SMR and proportion of dcSSc (*p* < 0.001) and prevalence of anti-Scl70 antibodies (*p* = 0.021). Our high SMR of 5.73 could therefore be partly explained by the high proportion of anti-Scl70 antibodies (35%) in our population. Interestingly, there has been a debate whether or not the survival could have improved over time in SSc. Our study did not show any improvement of SMR over time, which is in line with the study of Elhai et al. [6]. However, considering life expectancy in OECD (Organisation for Economic Co-operation and Development) countries increased by 12 years from 1960 to 2014 [56], and SMR being the ratio of mortality in SSc cohorts to that of the general population, this suggests that all-cause mortality has decreased proportionately to the general population in SSc cohorts.

### Prognosis factors

Prognosis factors have been assessed in many observational studies [7, 13–26] and have been recently reviewed. Our systematic review and meta-analysis, as well as two prior meta-analyses [5, 27] and a recent EUSTAR study [2], have identified the following characteristics as consistently associated with a worse prognosis: male gender; older age; dcSSc; lung and cardiac involvement, including PH and ILD; kidney involvement; and inflammation. These robust factors are included in a recent prognosis score [2] as well as in older ones [57, 58].

Besides these well-known prognosis factors, our cohort study identifies new ones: telangiectasia, 6MWD, valvular disease, cancer, and autoantibody status.

Telangiectasia was slightly associated with a higher mortality in our study population. In contrast, Poormoghim et al. [51] reported a non-significant, yet elevated HR of 1.44 in a smaller cohort of Iranian patients. An increased number of telangiectasia has been suggested to be a clinical marker of microvascular disease in SSc and is associated with an increased risk of PAH [59].

The 6MWD is a simple tool used to assess submaximal functional capacity. It is influenced by various disease parameters during SSc and lacks organ specificity [60]. While the 6MWD has been shown to be an independent predictor of mortality in idiopathic PAH, its prognosis value in SSc-PAH is less clear [61]. To our knowledge, we are the first to report a negative association of the 6MWD with survival (HR = 0.51) in SSc patients. This can be at least partly explained by the association between the 6MWD and PAH [60].

While cardiac involvement in SSc patients is robustly associated with a poor prognosis, conferring a nearly fivefold increased risk of mortality in our meta-analysis, no study had yet focused on the valvular manifestations of SSc. A recent article comparing echocardiography in SSc patients and a matched control population showed a greater frequency of valvular regurgitation and valvular replacement due to regurgitation [62]. Moreover, in a large multicenter French cohort, De Groote et al. [63] reported 6.7% mitral regurgitation and 2.5% aortic regurgitations. To our knowledge, we are the first to describe an association between valvular disease and survival in SSc. These data indicate that more attention should be paid to valvular disease in SSc patients and further studies are needed to confirm its prognostic significance.

An increased incidence of malignancy has been reported during SSc, especially lung and hematological cancer [64]. Cancer has also been described among the leading cause of non-SSc-related deaths [7, 11, 15, 65], and a temporal relation has been reported between the onset of cancer and SSc [66]. As expected, in our cohort as well as in the meta-analysis, malignancy was significantly associated with shorter survival.

In our cohort, we did not observe any association between anti-Scl70 antibodies or ACA and survival. Interestingly, the absence of association of anti-Scl70 antibodies with survival has been suggested by numerous studies [7, 17, 18, 23, 25] while the protective role of anti-centromere antibodies is better established [7, 18, 22, 25, 48]. Our meta-analysis confirms that the presence of ACA is associated with better survival (pooled HR = 0.58). Moreover, our meta-analysis suggests that the presence of anti-Scl70 antibodies could be indeed a predictor of mortality with a pooled HR of 1.38. Our analysis also highlights a probable publication bias. Small studies reporting a negative association of anti-Scl70 antibodies with death are notably lacking. Therefore, it is difficult to draw a firm conclusion on the role of anti-Scl70 antibodies as a prognosis factor in SSc.

The major strength of our study is the availability of detailed clinical and laboratory characteristics in a multicenter cohort of incident patients. The major strengths of our meta-analysis include (i) the first analysis of pooled HR of anti-Scl70/ACA antibodies, (ii) the separate analysis of pooled SMR in incident cohorts of SSc, and (iii) the separate analysis of pooled HR of PH diagnosed by RHC only.

The main limitation is a proportion of loss to follow-up of around 20% in our cohort, despite our attempts to collect information on participants who dropped out. These patients lost to follow-up had a higher prevalence of ILD and lower prevalence of ACA at baseline, leading to potential underestimation of mortality. A second limitation is the variable definition of incident patients among studies. Although we defined incident patients as newly diagnosed ones, these patients had relatively short disease duration from first non-RP symptom diagnosis, thus minimizing survivor bias. Because of missing values, the effect of specific treatments and other data such as type and stage of cancer could not be studied, and no multivariate analysis could be performed. In addition, multiple univariate tests are responsible for an inflation of the alpha risk. However, multiple test adjustments in such exploratory study, in addition for a rare disease, are not strictly required [67]. Finally, our study was performed in five selected referral centers and may therefore have focused on a subset of patients with more severe disease, which could limit the representativeness of our findings.

## Conclusions

Our results show that mortality is still high in SSc. Strong prognosis factors identified at baseline are age at diagnosis > 60 years, dcSSc subtype, scleroderma renal crisis, severe dyspnea, FVC and DLCO < 70%, PH, anemia, and CRP > 8 mg/l. Our study also suggests the prognosis value of telangiectasia, 6MWD, valvular disease, cancer, and autoantibody status.

## Additional files

Additional file 1: ** Table S1.** Survival rates and survival curve for lcSSc and dcSSc. Table S2. Main characteristics of studies in the SMR meta-analysis. Table S3. Main characteristics of studies in the prognosis factors meta-analysis. (DOCX 51 kb)

Additional file 2: ** Figure S1. **Funnel plots, forest plots, and meta-regression for the meta-analysis of SMR. Figure S2. Funnel plots and forest plots of the risk factors related with mortality. (PDF 948 kb)

## Abbreviations

6MWD: 6-min walking distance
ACA: Anti-centromere antibodies
APL: Antiphospholipid antibodies
AV block: Atrioventricular block
BB block: Bundle branch block
BMI: Body mass index
CPK: Creatinine kinase
CRP: C reactive protein
dcSSc: Diffuse cutaneous systemic sclerosis
DLCO: Diffusing capacity of the lungs for carbon monoxide
FVC: Forced vital capacity
ILD: Interstitial lung disease
lcSSc: Limited cutaneous systemic sclerosis
LVEF: Left ventricular ejection fraction
mRSS: Modified Rodnan skin score
OECD: Organisation for Economic Co-operation and Development
PAH: Pulmonary arterial hypertension
PAP: Pulmonary arterial pressure
PH: Pulmonary hypertension
RHC: Right heart catheterization
RP: Raynaud phenomenon
SMR: Standardized mortality ratio
SSc: Systemic sclerosis
TLC: Total lung capacity
