# Supplementary material for: Survival and prognosis factors in systemic sclerosis: data of a French multicenter cohort, systematic review, and meta-analysis of the literature
Source: Arthritis Res Ther. 2019 Apr 3;21:86. doi: 10.1186/s13075-019-1867-1 (PMC6446383; doi:10.1186/s13075-019-1867-1)
Supplement: Supplementary file 1 — Table S1. Survival rates and survival curve for lcSSc and dcSSc. Table S2. Main characteristics of studies in the SMR meta-analysis. Table S3. Main characteristics of studies in the prognosis factors meta-analysis. (DOCX 51 kb) [file 13075_2019_1867_MOESM1_ESM.docx]

**Additional file 1**

Supplementary Table 1. Survival rates and survival curve for lcSSc and dcSSc

Supplementary Table 2. Main characteristics of studies in the SMR meta-analysis

Supplementary Table 3. Main characteristics of studies in the prognosis factors meta-analysis

**Supplementary Table 1. Survival rates and survival curve for lcSSc and dcSSc**

|  | **1-year  survival rate** | **3-year  survival rate** | **5-year  survival rate** | **10-year  survival rate** |
| --- | --- | --- | --- | --- |
| **Overall** | 98.0 (96.9-99.1) | 92.5  (90.4-94.7) | 85.9 (82.8-89.1) | 71.7 (66.3-77.5) |
| **lcSSc** | 98.2  (96.9-99.4) | 94.0  (91.7-96.4) | 88.2  (84.8-91.7) | 75.9  (69.7-82.7) |
| **dcSSc** | 97.1 (94.7-99.6) | 88.0  (83.1-93.3) | 78.9  (72.1-86.3) | 57.0  (45.8-70.9) |

Results are given as % (95%CI)

lcSSc: diffuse cutaneous systemic sclerosis, dcSSc: diffuse cutaneous systemic sclerosis

**Supplementary Table 2. Main characteristics of studies in the SMR meta-analysis**

| First author | Study period | Mid-cohort year | Country | n | Inclusion of incident cases only | Study type | SMR | Males (%) | dc-SSc (%) | ACA (%) | Anti-Scl70 (%) | NOS rating |
| --- | --- | --- | --- | --- | --- | --- | --- | --- | --- | --- | --- | --- |
| Abu-Shakra [33] | 1979-1990 | 1984.5 | Canada | 237 | No | Prospective | 4.69 (3.58-6.02) | 17.3 | 43.0 | NA | NA | 7 |
| Alamanos [8] | 1981-2002 | 1991.5 | Greece | 109 | Yes | Retrospective | 2.00 (1.20-2.80) | 10.1 | 24.8 | NA | NA | 8 |
| Alba [24] | 2006-2012 | 2009 | Spain | 1037 | No | Retrospective | 3.80 (3.18-4.43) | 12.5 | 26.1 | 43.6 | 22.2 | 8 |
| Bryan [9] | 1982-1992 | 1987 | UK | 283 | Yes | Retrospective | 4.05 (3.03-5.22) | 23.0 | 45.9 | NA | NA | 8 |
| Cruz-Domínguez [34] | 2005-2014 | 2009.5 | Mexico | 220 | No | Retrospective | 4.50 (2.99-6.50) | 9.5 | 57.3 | 29.1 | 22.3 | 7 |
| Hao [7] | 2007-2014 | 2010.5 | Australia | 389 | Yes | Prospective | 3.40 (2.30-4.50) | 18.3 | 40.2 | 37.4 | 18.7 | 7 |
|  | 2007-2014 | 2010.5 | Australia | 1411 | No | Prospective | 2.80 (2.40-3.30) | 9.5 | 26.3 | 46.4 | 14.5 |  |
|  | 2005-2014 | 2009.5 | Canada | 484 | Yes | Prospective | 5.10 (4.00-6.20) | 19.2 | 44.0 | 29.5 | 18.2 |  |
|  | 2005-2014 | 2009.5 | Canada | 1465 | No | Prospective | 3.80 (3.30-4.20) | 14.1 | 36.2 | 34.5 | 15.2 |  |
|  | 2000-2014 | 2007 | Spain | 197 | Yes | Prospective | 3.20 (2.30-4.20) | 12.2 | 31.4 | 42.3 | 27.0 |  |
|  | 2000-2014 | 2007 | Spain | 342 | No | Prospective | 4.20 (3.30-5.00) | 13.7 | 30.4 | 41.9 | 26.9 |  |
| Hesselstrand [35] | 1983-1995 | 1989 | Sweden | 249 | No | Prospective | 4.59 (3.48-6.07) | 28.5 | 25.3 | NA | NA | 8 |
| Hoffmann- Vold [22] | 1999-2009 | 2004 | Norway | 312 | No | Retrospective | 2.03 (1.40-2.60) | 22.8 | 21.8 | NA | NA | 8 |
| Ioannidis [10] | NA | 1989 | Greece | 84 | Yes | Retrospective | 2.77 (1.33-5.87) | NA | NA | NA | NA | 7 |
|  | NA | 1989 | Japan | 156 | Yes | Retrospective | 3.20 (2.33-4.41) | NA | NA | NA | NA |  |
|  | NA | 1989 | Nether-lands /Leiden | 53 | Yes | Retrospective | 7.18 (4.31-11.96) | NA | NA | NA | NA |  |
|  | NA | 1989 | Nether-lands/ Nijmegen | 69 | Yes | Retrospective | 7.18 (4.94-10.53) | NA | NA | NA | NA |  |
|  | NA | 1989 | USA /Mayo | 105 | Yes | Retrospective | 1.50 (1.06-2.12) | NA | NA | NA | NA |  |
| Jacobsen [11] | 1960-1996 | 1978 | Denmark | 344 | Yes | Retrospective | 2.90 (2.50-3.40) | 19.2 | 34.3 | NA | NA | 8 |
| Kuo [12] | 2002-2007 | 2004.5 | Taiwan | 1479 | Yes | Retrospective | 3.24 (2.82-3.71) | 22.0 | NA | NA | NA | 8 |
| Mok [36] | 1999-2008 | 2003.5 | China | 449 | No | Retrospective | 3.94 (3.20-4.68) | 14.9 | NA | NA | NA | 8 |
| Nihtyanova [26] | 1995-2010 | 2002.5 | UK | 398 | Yes | Retrospective | 3.82 (3.13-4.52) | 13.6 | 36.7 | 27.7 | 21.3 | 8 |
| Pérez- Bocanegra [37] | 1976-2007 | 1991.5 | Spain | 319 | No | Prospective | 1.90 (1.50-2.30) | NA | 20.1 | 42.6 | 19.0 | 6 |
| Pokeerbux | 2000-2016 | 2008 | France | 625 | Yes | Retrospective | 5.73 (4.68-6.94) | 21.1 | 28.6 | 39.7 | 35.1 | 7 |
| Scussel- Lonzetti [15] | 1984-1999 | 1991.5 | Canada | 309 | No | Prospective | 2.69 (2.10-3.40) | 13.9 | 34.6 | 43.7 | 12.0 | 8 |
| Strickland [23] | 1999-2010 | 2004.5 | UK | 204 | No | Retrospective | 1.34 (1.00-1.75) | 12.3 | 19.6 | 47.1 | 16.2 | 8 |
| Zarafonetis [38] | 1948-1980 | 1964 | USA | 390 | No | Retrospective | 5.40 (4.55-6.37) | 18.5 | NA | NA | NA | 7 |

N : number of patients, dcSSc: diffuse cutaneous systemic sclerosis, ACA: anti-centromere antibodies, anti-Scl70: anti-Scl70 antibodies, NOS: Newcastle-Ottawa scale

**Supplementary Table 3. Main characteristics of studies in the prognosis factors meta-analysis**

| First author | Study period | Mid-cohort year | Country | n | Inclusion  of incident cases only | Study type | Males (%) | dcSSc (%) | ACA (%) | Anti-Scl70 (%) | NOS rating |
| --- | --- | --- | --- | --- | --- | --- | --- | --- | --- | --- | --- |
| Alba [24] | 2006-2012 | 2009 | Spain | 1037 | No | Retrospective | 12.5 | 30.2 | 43.6 | 22.2 | 8 |
| Al-Dhaher [3] | 1994-2004 | 1999 | Canada | 185 | No | Retrospective | 14.6 | 36.8 | NA | NA | 7 |
| Assassi [18] | 1998-2005 | 2001.5 | USA | 250 | Yes | Prospective | 16.0 | 57.2 | 11.7 | 18.9 | 8 |
| Beretta [39] | 1982-2008 | 1995 | Italia | 558 | No | Retrospective | 10.6 | 26.9 | 34.2 | 43.7 | 6 |
| Beretta [40] | 1997-2005 | 2001 | Italia | 161 | No | Retrospective | 12.4 | 28.6 | 44.7 | 40.4 | 7 |
| Bernal-Bello [41] | 1980-2014 | 1997 | Spain | 432 | No | Retrospective | 11.3 | 21.3 | 41.6 | 20.9 | 7 |
| Codullo [42] | 2006-2012 | 2009 | Italia | 299 | No | Retrospective | 13.0 | 17.1 | 54.8 | 16.4 | 6 |
| Cottrell [25] | 1976-2010 | 1993 | USA | 2205 | No | Retrospective | 17.0 | 38.9 | 27.5 | 23.0 | 7 |
| Cruz-Dominguez [34] | 2005-2014 | 2009.5 | Mexico | 220 | No | Retrospective | 9.5 | 57.3 | 29.1 | 22.3 | 7 |
| Czirjak [16] | 1983-2005 | 1994 | Hungary | 366 | No | NA | 13.9 | 27.6 | 13.1 | 36.6 | 7 |
| Fernandez-Codina [43] | 1976-2011 | 1993.5 | Spain | 393 | No | Retrospective | 10.4 | 19.3 | 40.0 | 18.0 | 7 |
| Ferri [44] | 2000-2011 | 2005.5 | Italia | 821 | No | Retrospective | 9.1 | 12.5 | NA | NA | 8 |
| Ferri [14] | 1955-1999 | 1977 | Italia | 1012 | No | Retrospective | 11.4 | 44.0 | 38.9 | 36.0 | 8 |
| Gelber [45] | 1990-2009 | 1999.5 | USA | 2217 | No | Prospective | 17.1 | NA | 29.9 | 21.1 | 8 |
| Hachulla [17] | 2002-2005 | 2003.5 | France | 546 | No | Prospective | 15.9 | 27.5 | 48.1 | 28.0 | 7 |
| Hao [7] | 2000-2014 | 2007 | Australia, Canada, Spain | 1070 | Yes | Retrospective | 17.6 | 40.9 | 35.4 | 20.3 | 7 |
|  | 2000-2014 | 2007 | Australia, Canada, Spain | 3218 | No | Retrospective | 13.6 | 32.5 | 40.9 | 16.3 |  |
| Hesselstrand [46] | 1983-1998 | 1990.5 | Sweden | 276 | No | NA | 26.1 | 24.6 | 18.5 | 9.4 | 8 |
| Hinchcliff [47] | 2005-2009 | 2007 | USA | 153 | No | Retrospective | 15.0 | 39.9 | NA | NA | 8 |
| Hoffmann-Vold [22] | 1999-2009 | 2004 | Norway | 312 | No | Retrospective | 22.8 | 21.8 | NA | NA | 7 |
| Hussein [48] | 1970-2013 | 1991.5 | Canada | 959 | No | Retrospective | 17.5 | 32.5 | NA | NA | 8 |
| Ioannidis [10] | NA | 1989 | International | 467 | Yes | Retrospective | NA | NA | NA | NA | 7 |
| Jacobsen [11] | 1960-1996 | 1978 | Denmark | 174 | Yes | Retrospective | 16.1 | 32.8 | 37.0 | 13.0 | 8 |
| Kim [20] | 1972-2007 | 1989.5 | Korea | 230 | No | Retrospective | 10.9 | 43.9 | 13.6 | 53.6 | 8 |
| Költő [49] | 2007-2012 | 2009.5 | Hungary | 120 | No | Retrospective | 11.7 | 32.5 | NA | NA | 8 |
| Kuo [12] | 2002-2007 | 2004.5 | Taiwan | 1479 | Yes | Retrospective | 22.0 | NA | NA | NA | 8 |
| Lee [13] | 1979-1990 | 1984.5 | Canada | 237 | No | Prospective | 17.3 | 43.0 | NA | NA | 7 |
| Mayes [50] | 1989-1991 | 1990 | USA | 706 | No | Retrospective | 16.3 | 34.9 | 22.1 | 19.6 | 8 |
| Nihtyanova [26] | 1995-2010 | 2002.5 | UK | 398 | Yes | Retrospective | 13.6 | 36.7 | 27.7 | 21.3 | 8 |
| Pokeerbux | 2000-2016 | 2008 | France | 625 | Yes | Retrospective | 21.1 | 28.6 | 39.7 | 35.1 | 7 |
| Poormoghim [51] | 1998-2012 | 2005 | Iran | 220 | No | Prospective | 12.7 | 40.0 | 8.4 | 70.2 | 8 |
| Ruangjutipopan [52] | 1987-2001 | 1994 | Thailand | 222 | No | Retrospective | 23.8 | 57.2 | NA | NA | 6 |
| Scussel-Lonzetti [15] | 1984-1999 | 1991.5 | Canada | 309 | No | Prospective | 14.0 | 34.6 | 43.7 | 12.0 | 8 |
| Simeon [53] | 1976-1996 | 1986 | Spain | 79 | Yes | Retrospective | 13.9 | 27.8 | NA | NA | 6 |
| Simeon-Aznar [54] | 2006-2008 | 2007 | Spain | 879 | No | Retrospective | 14.8 | 27.6 | NA | NA | 8 |
| Steen [55] | 1972-2007 | 1989.5 | USA | 3148 | No | Prospective | 19.7 | 43.4 | 21.1 | 17.5 | 8 |
| Strickland [23] | 1999-2010 | 2004.5 | UK | 204 | No | Retrospective | 12.3 | 19.6 | 47.1 | 16.2 | 8 |

N : number of patients, dcSSc: diffuse cutaneous systemic sclerosis, ACA: anti-centromere antibodies, anti-Scl70: anti-Scl70 antibodies, NOS: Newcastle-Ottawa scale
