# Supplementary material for: Survival and prognosis factors in systemic sclerosis: data of a French multicenter cohort, systematic review, and meta-analysis of the literature
Source: Arthritis Res Ther. 2019 Apr 3;21:86. doi: 10.1186/s13075-019-1867-1 (PMC6446383; doi:10.1186/s13075-019-1867-1)
Supplement: Supplementary file 2 — Figure S1. Funnel plots, forest plots, and meta-regression for the meta-analysis of SMR. Figure S2. Funnel plots and forest plots of the risk factors related with mortality. (PDF 948 kb) [file 13075_2019_1867_MOESM2_ESM.pdf]

Additional file 2

Supplementary Figure 1. Funnel plots, forest plots and meta-regression for the meta-analysis of SMR

Supplementary Figure 2. Funnel plots and forest plots of the risk factors related with mortality

Supplementary figure 1: funnel plots, forest plots and meta-regression for the meta-analysis of SMR

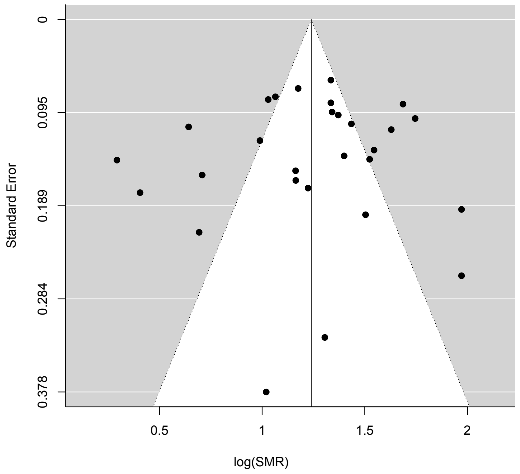

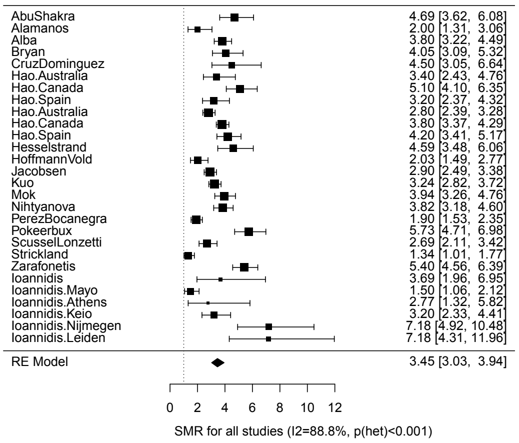

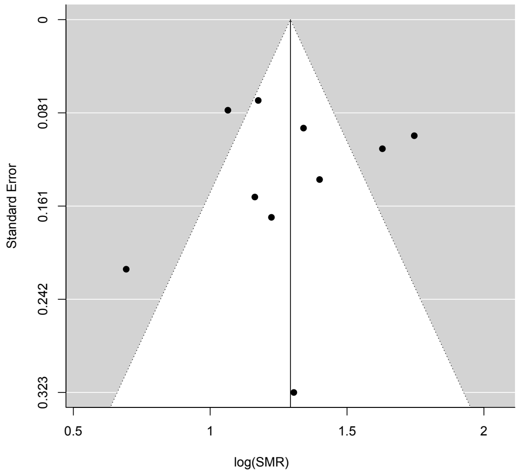

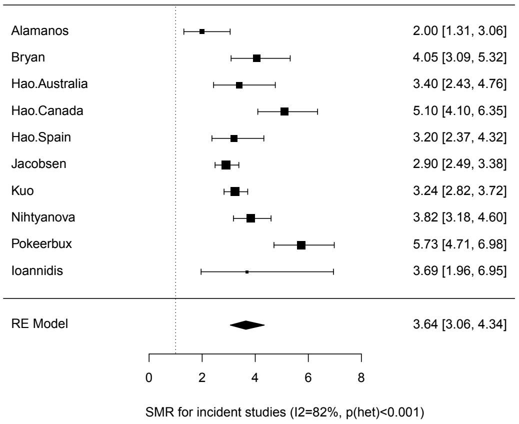

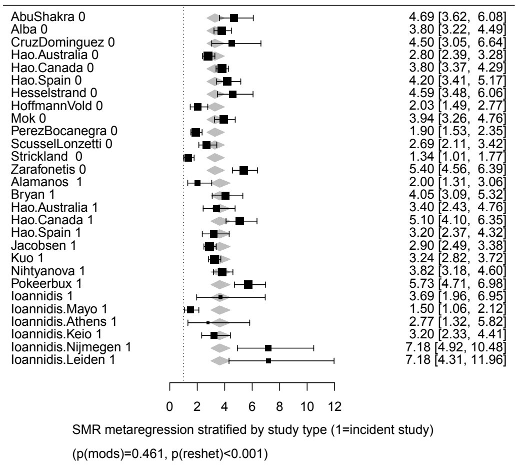

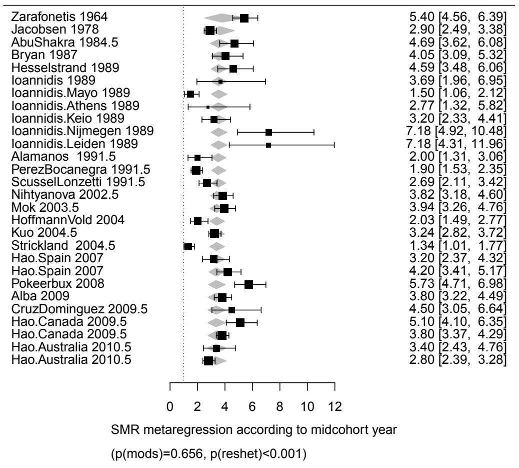

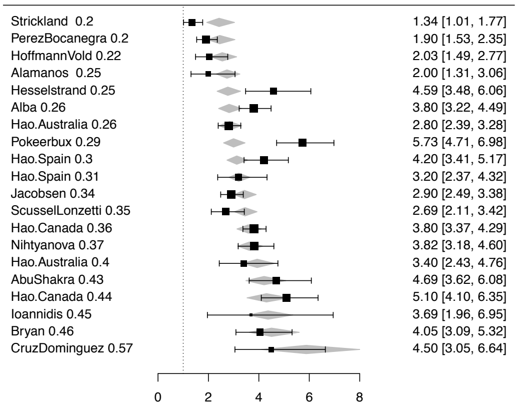

SMR metaregression according to proportion of dcSSc

(p(mods)<0.001, p(reshet)<0.001)

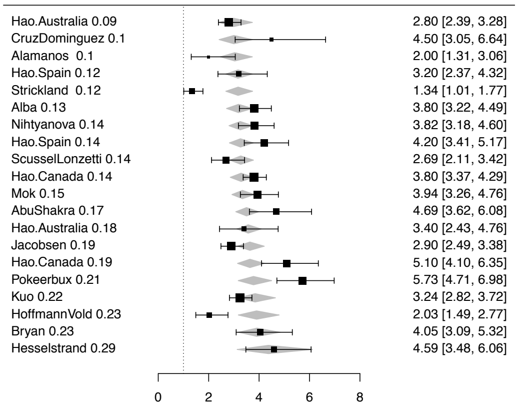

SMR metaregression according to proportion of males

(p(mods)=0.130, p(reshet)<0.001)

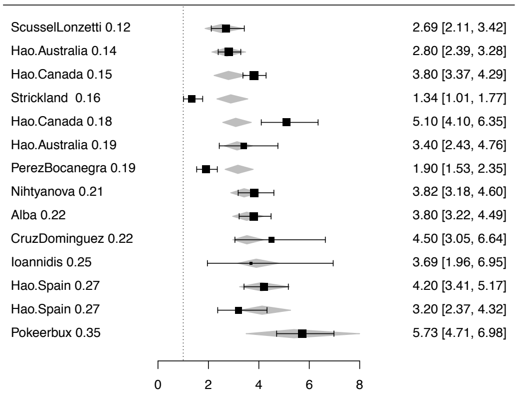

SMR metaregression according to proportion of anti-Scl70 antibodies

(p(mods)=0.021, p(reshet)<0.001)

Footnote:
p(het): p for heterogeneity between studies
p(reshet): p for residual heterogeneity between studies
p(mods): p for moderator effect

dcSSc: diffuse cutaneous systemic sclerosis

Supplementary figure 2: funnel plots and forest plots of the risk factors related with mortality

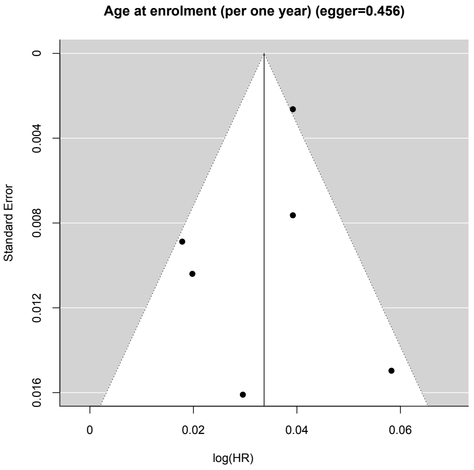

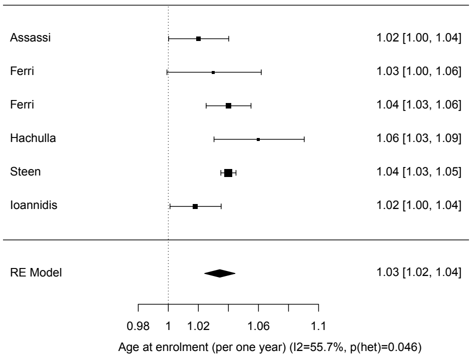

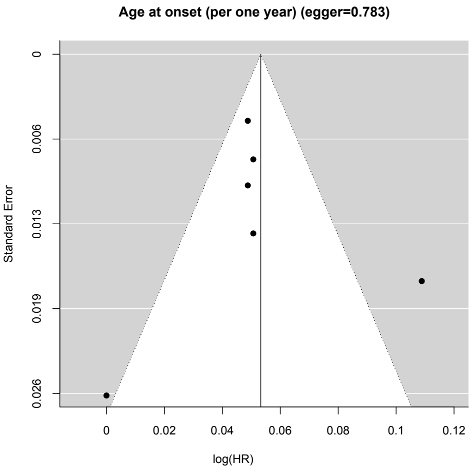

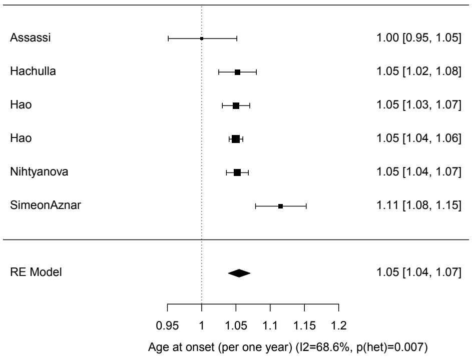

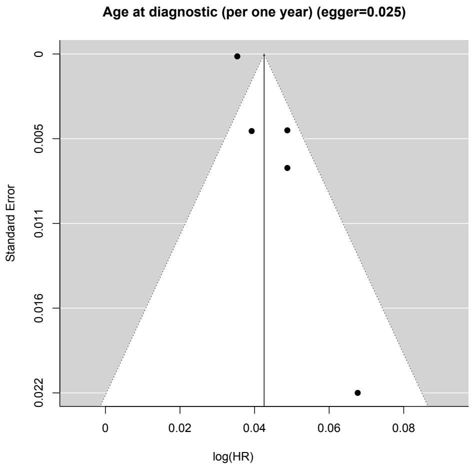

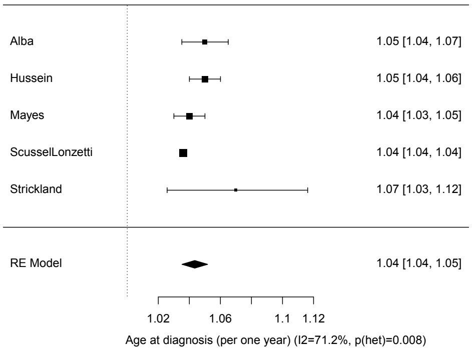

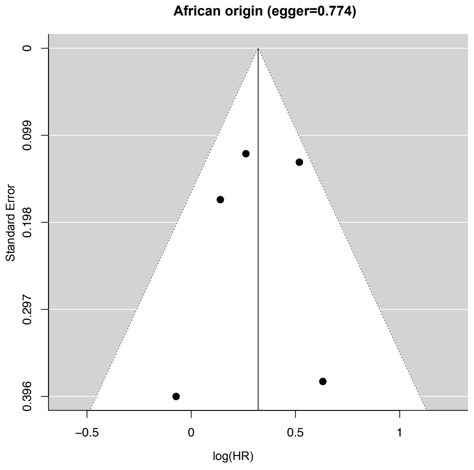
African origin (egger=0.774)
Standard Error
0
0.099
0.198
0.297
0.396
−0.5
0
0.5
1
log(HR)

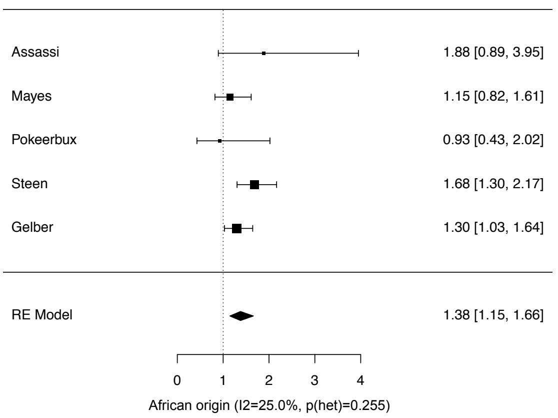
Assassi 1.88 [0.89, 3.95]
Mayes 1.15 [0.82, 1.61]
Pokeerbux 0.93 [0.43, 2.02]
Steen 1.68 [1.30, 2.17]
Gelber 1.30 [1.03, 1.64]
RE Model 1.38 [1.15, 1.66]
0 1 2 3 4
African origin (I2=25.0%, p(het)=0.255)

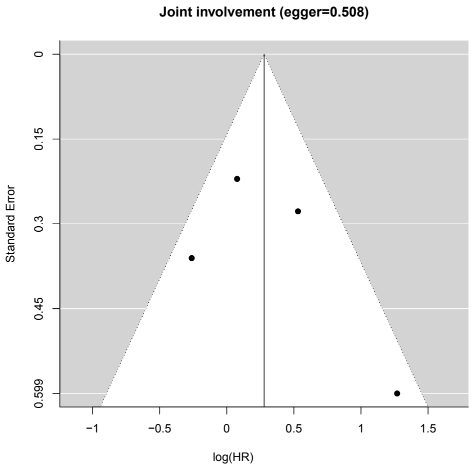
Joint involvement (egger=0.508)
Standard Error
0
0.15
0.3
0.45
0.599
−1
−0.5
0
0.5
1
1.5
log(HR)

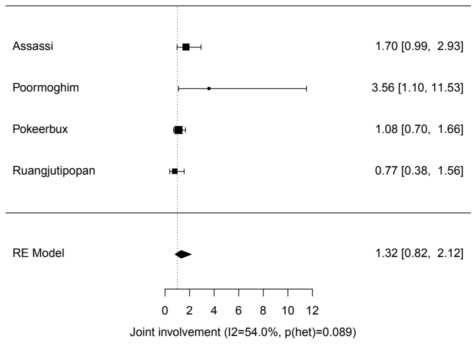
Assassi 1.70 [0.99, 2.93]
Poormoghim 3.56 [1.10, 11.53]
Pokeerbux 1.08 [0.70, 1.66]
Ruangjutipopan 0.77 [0.38, 1.56]
RE Model 1.32 [0.82, 2.12]
0 2 4 6 8 10 12
Joint involvement (I2=54.0%, p(het)=0.089)

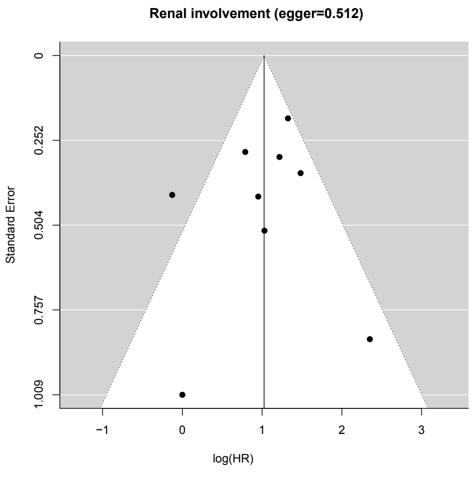
Renal involvement (egger=0.512)
Standard Error
0
0.252
0.504
0.757
1.009
−1
0
1
2
3
log(HR)

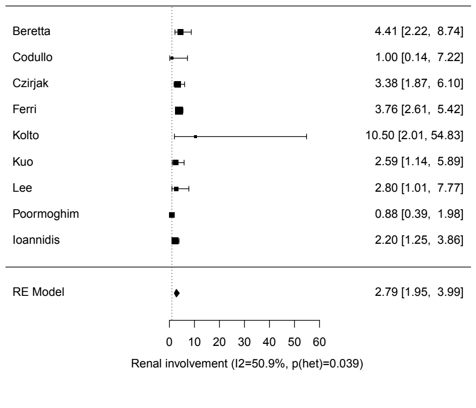
Beretta 4.41 [2.22, 8.74]
Codullo 1.00 [0.14, 7.22]
Czirjak 3.38 [1.87, 6.10]
Ferri 3.76 [2.61, 5.42]
Kolto 10.50 [2.01, 54.83]
Kuo 2.59 [1.14, 5.89]
Lee 2.80 [1.01, 7.77]
Poormoghim 0.88 [0.39, 1.98]
Ioannidis 2.20 [1.25, 3.86]
RE Model 2.79 [1.95, 3.99]
0 10 20 30 40 50 60
Renal involvement (I2=50.9%, p(het)=0.039)

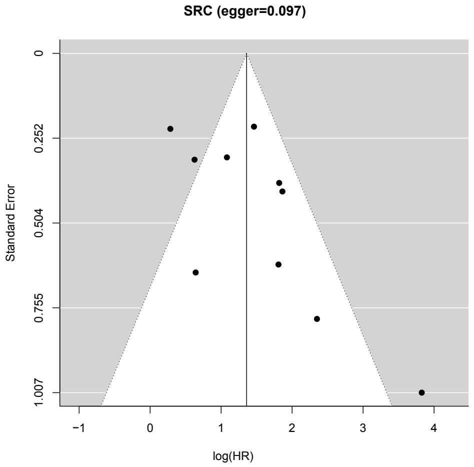
SRC (egger=0.097)
Standard Error
0
0.252
0.504
0.755
1.007
−1 0 1 2 3 4
log(HR)

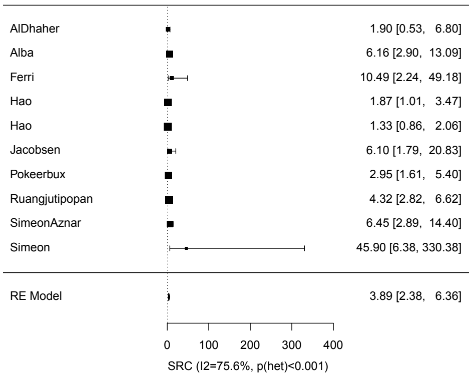
AlDhaher 1.90 [0.53, 6.80]
Alba 6.16 [2.90, 13.09]
Ferri 10.49 [2.24, 49.18]
Hao 1.87 [1.01, 3.47]
Hao 1.33 [0.86, 2.06]
Jacobsen 6.10 [1.79, 20.83]
Pokeerbux 2.95 [1.61, 5.40]
Ruangjutipopan 4.32 [2.82, 6.62]
SimeonAznar 6.45 [2.89, 14.40]
Simeon 45.90 [6.38, 330.38]
RE Model 3.89 [2.38, 6.36]
0 100 200 300 400
SRC (I2=75.6%, p(het)<0.001)

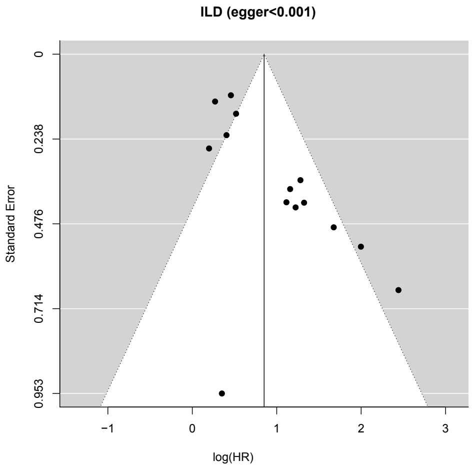
ILD (egger<0.001)
Standard Error
0
0.238
0.476
0.714
0.953
−1 0 1 2 3
log(HR)

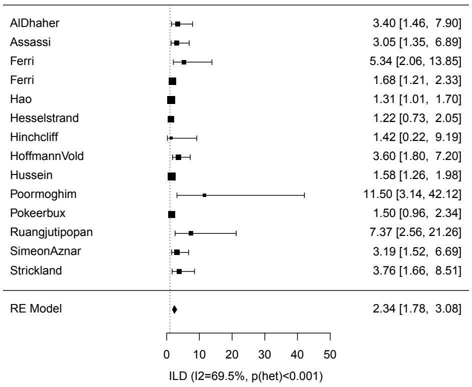
AlDhaher 3.40 [1.46, 7.90]
Assassi 3.05 [1.35, 6.89]
Ferri 5.34 [2.06, 13.85]
Ferri 1.68 [1.21, 2.33]
Hao 1.31 [1.01, 1.70]
Hesselstrand 1.22 [0.73, 2.05]
Hinchcliff 1.42 [0.22, 9.19]
HoffmannVold 3.60 [1.80, 7.20]
Hussein 1.58 [1.26, 1.98]
Poormoghim 11.50 [3.14, 42.12]
Pokeerbux 1.50 [0.96, 2.34]
Ruangjutipopan 7.37 [2.56, 21.26]
SimeonAznar 3.19 [1.52, 6.69]
Strickland 3.76 [1.66, 8.51]
RE Model 2.34 [1.78, 3.08]
0 10 20 30 40 50
ILD (I2=69.5%, p(het)<0.001)

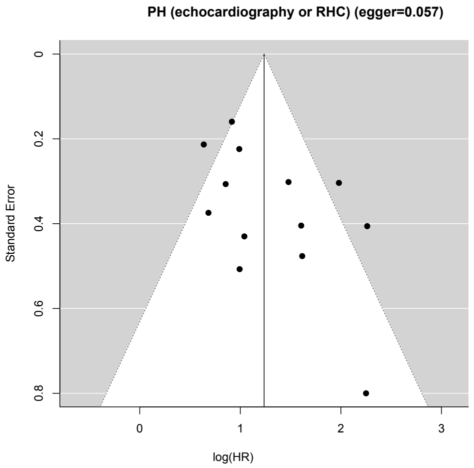
PH (echocardiography or RHC) (egger=0.057)
Standard Error
0
0.2
0.4
0.6
0.8
0 1 2 3
log(HR)

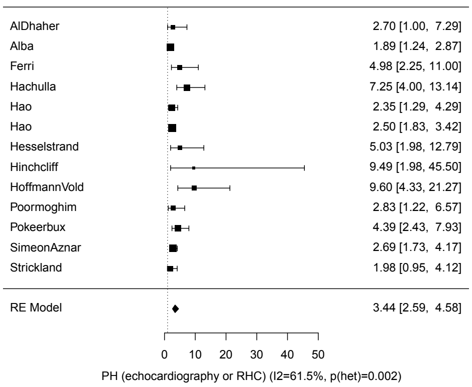
AlDhaher 2.70 [1.00, 7.29]
Alba 1.89 [1.24, 2.87]
Ferri 4.98 [2.25, 11.00]
Hachulla 7.25 [4.00, 13.14]
Hao 2.35 [1.29, 4.29]
Hao 2.50 [1.83, 3.42]
Hesselstrand 5.03 [1.98, 12.79]
Hinchcliff 9.49 [1.98, 45.50]
HoffmannVold 9.60 [4.33, 21.27]
Poormoghim 2.83 [1.22, 6.57]
Pokeerbux 4.39 [2.43, 7.93]
SimeonAznar 2.69 [1.73, 4.17]
Strickland 1.98 [0.95, 4.12]
RE Model 3.44 [2.59, 4.58]
0 10 20 30 40 50
PH (echocardiography or RHC) (I2=61.5%, p(het)=0.002)

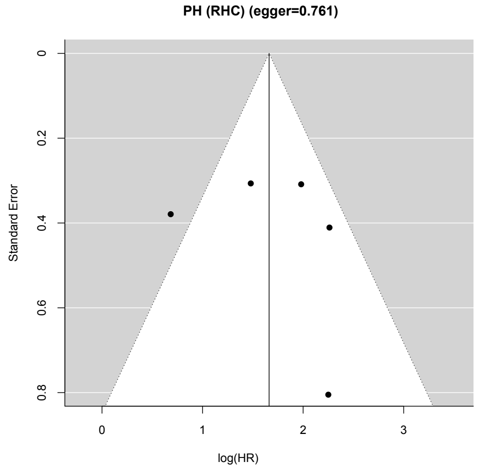
PH (RHC) (egger=0.761)
Standard Error
0
0.2
0.4
0.6
0.8
0
1
2
3
log(HR)

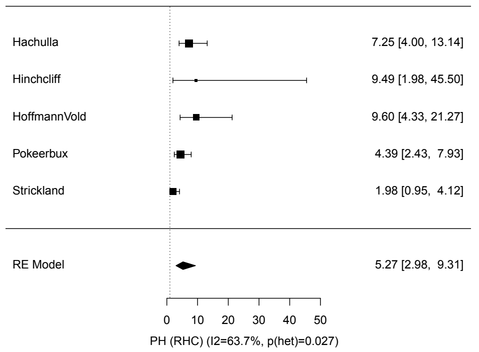
Hachulla 7.25 [4.00, 13.14]
Hinchcliff 9.49 [1.98, 45.50]
HoffmannVold 9.60 [4.33, 21.27]
Pokeerbux 4.39 [2.43, 7.93]
Strickland 1.98 [0.95, 4.12]
RE Model 5.27 [2.98, 9.31]
0 10 20 30 40 50
PH (RHC) (I2=63.7%, p(het)=0.027)

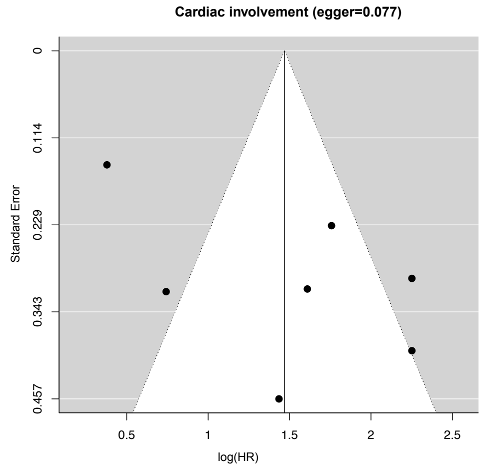
Cardiac involvement (egger=0.077)
Standard Error
0
0.114
0.229
0.343
0.457
0.5
1
1.5
2
2.5
log(HR)

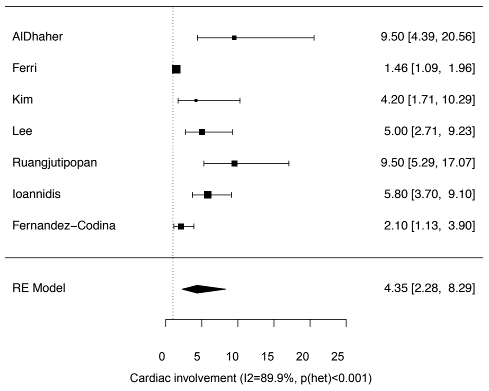
AlDhaher 9.50 [4.39, 20.56]
Ferri 1.46 [1.09, 1.96]
Kim 4.20 [1.71, 10.29]
Lee 5.00 [2.71, 9.23]
Ruangjutipopan 9.50 [5.29, 17.07]
Ioannidis 5.80 [3.70, 9.10]
Fernandez–Codina 2.10 [1.13, 3.90]
RE Model 4.35 [2.28, 8.29]
0 5 10 15 20 25
Cardiac involvement (I2=89.9%, p(het)<0.001)

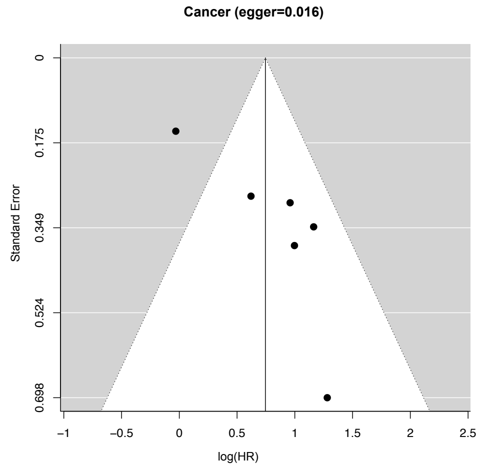
Cancer (egger=0.016)
Standard Error
0
0.175
0.349
0.524
0.698
−1
−0.5
0
0.5
1
1.5
2
2.5
log(HR)

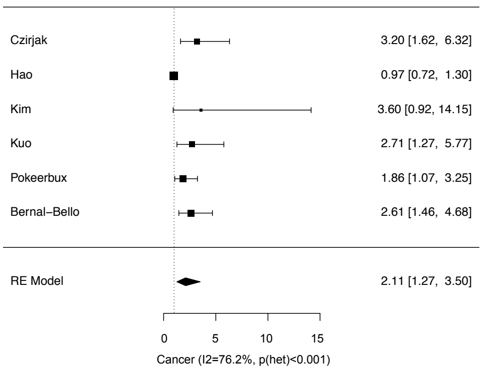
Czirjak 3.20 [1.62, 6.32]
Hao 0.97 [0.72, 1.30]
Kim 3.60 [0.92, 14.15]
Kuo 2.71 [1.27, 5.77]
Pokeerbux 1.86 [1.07, 3.25]
Bernal–Bello 2.61 [1.46, 4.68]
RE Model 2.11 [1.27, 3.50]
0 5 10 15
Cancer (I2=76.2%, p(het)<0.001)

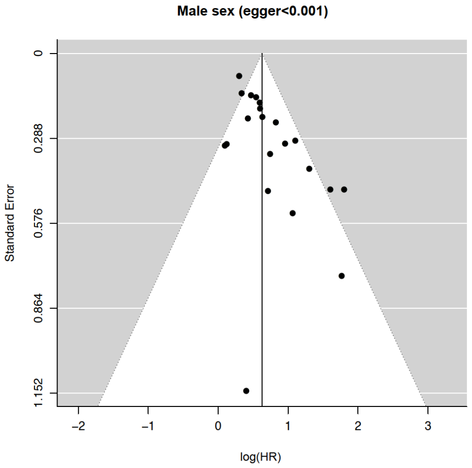
Male sex (egger<0.001)
Standard Error
0
0.288
0.576
0.864
1.152
−2
−1
0
1
2
3
log(HR)

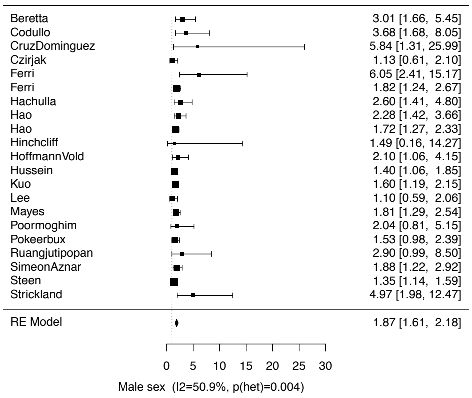
Beretta 3.01 [1.66, 5.45]
Codullo 3.68 [1.68, 8.05]
CruzDominguez 5.84 [1.31, 25.99]
Czirjak 1.13 [0.61, 2.10]
Ferri 6.05 [2.41, 15.17]
Ferri 1.82 [1.24, 2.67]
Hachulla 2.60 [1.41, 4.80]
Hao 2.28 [1.42, 3.66]
Hao 1.72 [1.27, 2.33]
Hinchcliff 1.49 [0.16, 14.27]
HoffmannVold 2.10 [1.06, 4.15]
Hussein 1.40 [1.06, 1.85]
Kuo 1.60 [1.19, 2.15]
Lee 1.10 [0.59, 2.06]
Mayes 1.81 [1.29, 2.54]
Poormoghim 2.04 [0.81, 5.15]
Pokeerbux 1.53 [0.98, 2.39]
Ruangjutipopan 2.90 [0.99, 8.50]
SimeonAznar 1.88 [1.22, 2.92]
Steen 1.35 [1.14, 1.59]
Strickland 4.97 [1.98, 12.47]
RE Model 1.87 [1.61, 2.18]
0 5 10 15 20 25 30
Male sex (I2=50.9%, p(het)=0.004)

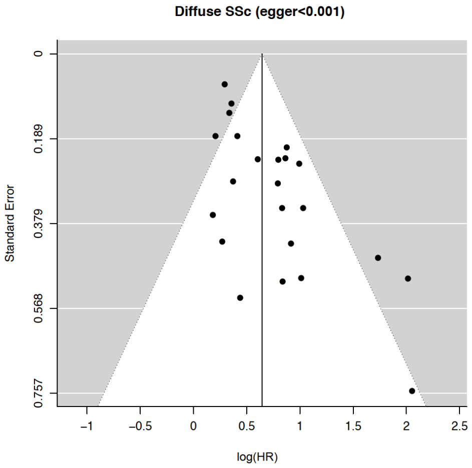
Diffuse SSc (egger<0.001)
Standard Error
0
0.189
0.379
0.568
0.757
−1
−0.5
0
0.5
1
1.5
2
2.5
log(HR)

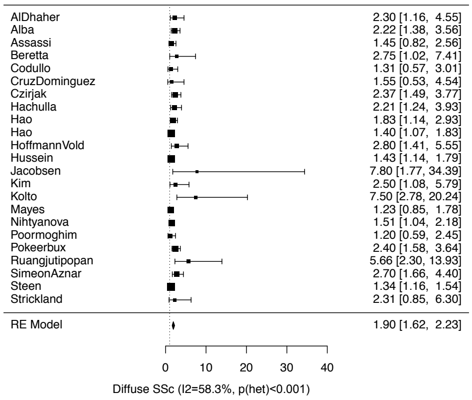
AlDhaher 2.30 [1.16, 4.55]
Alba 2.22 [1.38, 3.56]
Assassi 1.45 [0.82, 2.56]
Beretta 2.75 [1.02, 7.41]
Codullo 1.31 [0.57, 3.01]
CruzDominguez 1.55 [0.53, 4.54]
Czirjak 2.37 [1.49, 3.77]
Hachulla 2.21 [1.24, 3.93]
Hao 1.83 [1.14, 2.93]
Hao 1.40 [1.07, 1.83]
HoffmannVold 2.80 [1.41, 5.55]
Hussein 1.43 [1.14, 1.79]
Jacobsen 7.80 [1.77, 34.39]
Kim 2.50 [1.08, 5.79]
Kolto 7.50 [2.78, 20.24]
Mayes 1.23 [0.85, 1.78]
Nihtyanova 1.51 [1.04, 2.18]
Poormoghim 1.20 [0.59, 2.45]
Pokeerbux 2.40 [1.58, 3.64]
Ruangjutipopan 5.66 [2.30, 13.93]
SimeonAznar 2.70 [1.66, 4.40]
Steen 1.34 [1.16, 1.54]
Strickland 2.31 [0.85, 6.30]
RE Model 1.90 [1.62, 2.23]
0 10 20 30 40
Diffuse SSc (I2=58.3%, p(het)<0.001)

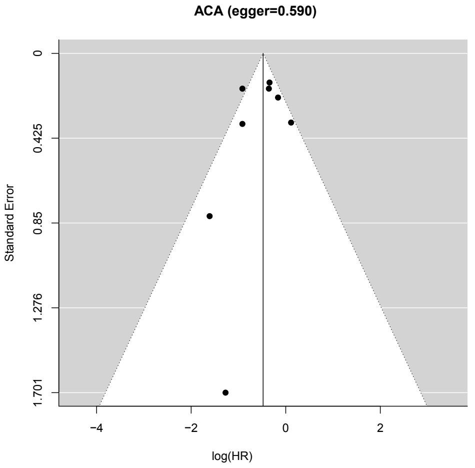

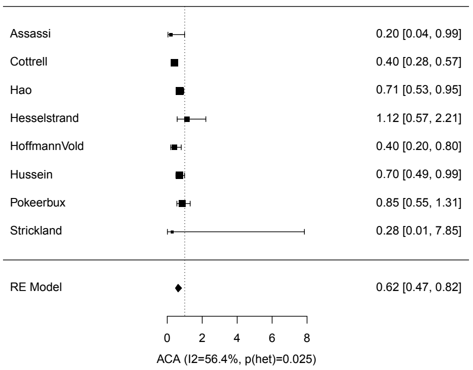

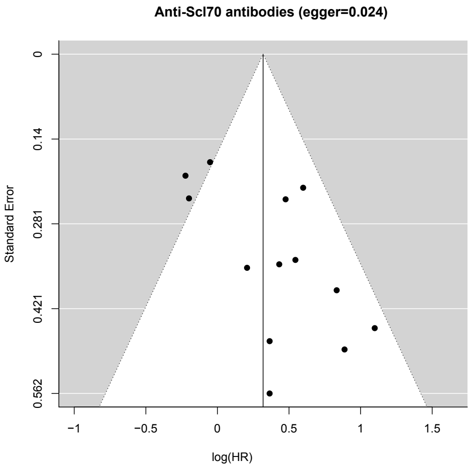

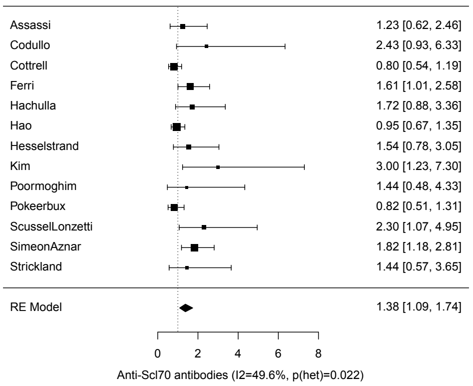

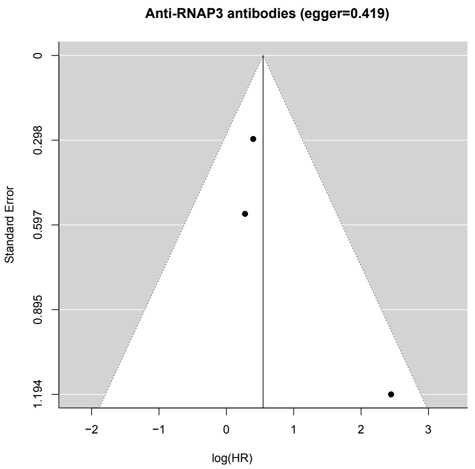

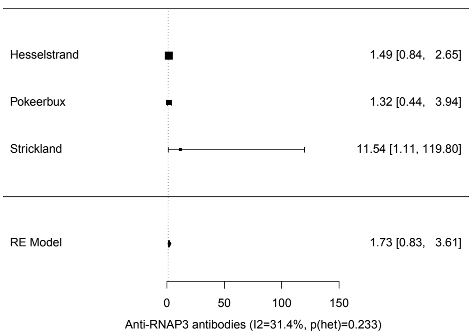

Footnote:
p(het): p for heterogeneity between studies
SRC: scleroderma renal crisis, ILD: interstitial lung disease, PH: pulmonary hypertension, RHC: right heart catheterization, ACA: anti-centromere antibodies
